# Potential Bioactive Function of Microbial Metabolites as Inhibitors of Tyrosinase: A Systematic Review

**DOI:** 10.3390/ijms27021016

**Published:** 2026-01-20

**Authors:** Sofia Barcenas-Giraldo, Vanessa Baez-Leguizamon, Laura Barbosa-Gonzalez, Angelica Leon-Rodriguez, Yovani Marrero-Ponce, Luis Diaz

**Affiliations:** 1 Master Program in Process Design and Management, School of Engineering, Universidad de La Sabana, Chía 140013, Colombia; paulabagi@unisabana.edu.co; 2 Bioprospecting Research Group, School of Engineering, Universidad de La Sabana, Chia 140013, Colombia; shirlybale@unisabana.edu.co (V.B.-L.); marialero@unisabana.edu.co (A.L.-R.); 3 Chemical Engineering Program, School of Engineering, Universidad de La Sabana, Chia 140013, Colombia; 4 Facultad de Ingeniería, Universidad Panamericana, Augusto Rodin No. 498, Insurgentes Mixcoac, Benito Juárez, Mexico City 03920, Mexico; ymarrero@up.edu.mx

**Keywords:** tyrosinase, microbial inhibitors, food browning, melanogenesis, whitening

## Abstract

Tyrosinase (EC 1.14.18.1) is a binuclear copper enzyme responsible for the rate-limiting steps of melanogenesis, catalyzing the hydroxylation of L-tyrosine and oxidation of L-DOPA into o-quinones that polymerize melanin. Beyond its physiological role in pigmentation, tyrosinase is also implicated in food browning and oxidative stress–related disorders, making it a key target in cosmetic, food, and biomedical industries. This systematic review, conducted following PRISMA guidelines, aimed to identify and analyze microbial metabolites with tyrosinase inhibitory potential as sustainable alternatives to conventional inhibitors such as hydroquinone and kojic acid. Literature searches in Scopus and Web of Science (March 2025) yielded 156 records; after screening and applying inclusion criteria, 11 studies were retained for analysis. The inhibitors identified include indole derivatives, phenolic acids, peptides, and triterpenoids, mainly produced by fungi (e.g., *Ganoderma lucidum*, *Trichoderma* sp.), actinobacteria (*Streptomyces*, *Massilia*), and microalgae (*Spirulina*, *Synechococcus*). Reported IC_50_ values ranged from micromolar to milli-molar levels, with methyl lucidenate F (32.23 µM) and *p*-coumaric acid (52.71 mM). Mechanisms involved competitive and non-competitive inhibition, as well as gene-level regulation. However, methodological heterogeneity, the predominance of mushroom tyrosinase assays, and limited human enzyme validation constrain translational relevance. Computational modeling, site-directed mutagenesis, and molecular dynamics are proposed to overcome these limitations. Overall, microbial metabolites exhibit promising efficacy, stability, and biocompatibility, positioning them as emerging preclinical candidates for the development of safer and more sustainable tyrosinase inhibitors.

## 1. Introduction

**Figure 1:**
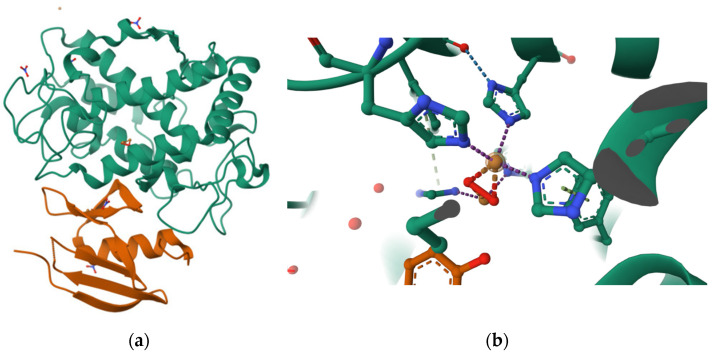
Overall structure and active site of *Streptomyces castaneoglobisporus* tyrosinase (PDB 1WX4). (**a**) Three-dimensional structure of tyrosinase in complex with the caddie protein showing the heterodimeric organization. (**b**) Detailed view of the catalytic pocket highlighting the binuclear copper center (CuA and CuB) coordinated by histidine residues, characteristic of type 3 copper enzymes [4].

**Figure 2:**
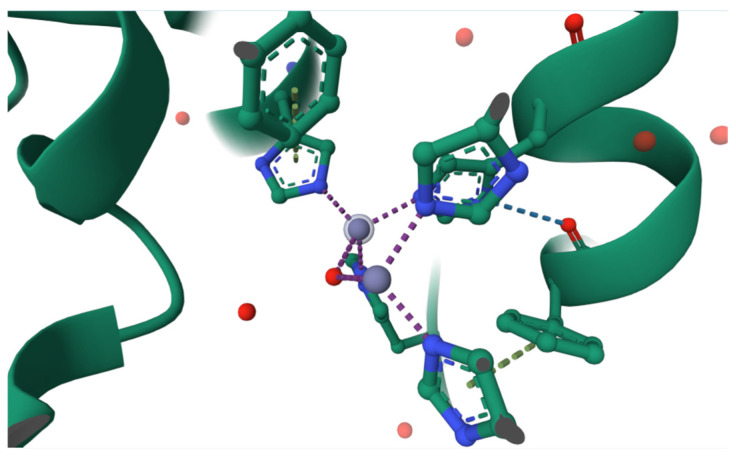
Active site of human tyrosinase-related protein 1 (TYRP1) (PDB ID: 5M8L). The catalytic pocket contains binuclear zinc ions (purple spheres) instead of copper, which are associated with reduced redox activity compared to tyrosinase and are linked to pigmentation disorders such as albinism [5].

Tyrosinase (EC 1.14.18.1), also called polyphenol oxidase or monophenol monooxygenase, is an enzyme characterized by two copper ions –CuA and CuB– on its active site which are coordinated by histidine residues forming a type 3 copper center, as shown in Figure 1. This particular active site composition eases the substrate–enzyme union and influences its integrity [1,2]. There are some human tyrosinase variations, for example TYRP1, in which the active site contains zinc instead of copper, as shown in Figure 2 where purple circles represent zinc ion in the active site; however, zinc has been involved in lower redox capacities resulting in albinism [3].

**Figure 3:**
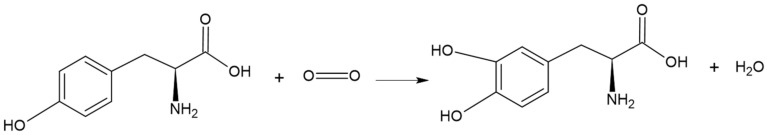
Tyrosine hydroxylation catalyzed by tyrosinase (monophenolase activity). Tyrosinase catalyzes the ortho-hydroxylation of L-tyrosine to form L-DOPA in the presence of molecular oxygen and copper ions at the active site [33].

**Figure 4:**
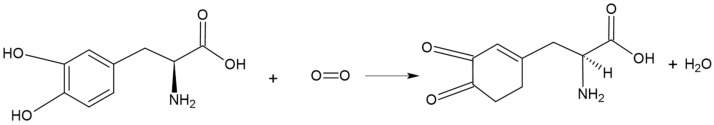
Oxidation of L-DOPA to dopaquinone catalyzed by tyrosinase (diphenolase activity). L-DOPA is oxidized to dopaquinone, a highly reactive o-quinone intermediate that initiates downstream melanogenesis reactions [33].

Tyrosinase is normally involved in the catalysis of one or both consecutive reactions to biosynthesize melanin. Transformation of L-tyrosine and L-dihydroxyphenylalanine (L-DOPA) into *o*-dopaquinone is conducted by a hydroxylation of monophenols followed by oxidation of *o*-quinone that is polymerized to melanin through melanogenesis [6,7], shown in Figure 3 and Figure 4.

Tyrosinase is present in a wide variety of organisms, including microorganisms, plants, and animals [7]. Gene expression control is dictated by cell type-specific regulatory sequences that physically organize chromatin structure, including promoters, enhancers, and insulators [8]. In mammals such as mice, a 5′ regulatory element of the *Tyr* gene has been described that acts as a distal enhancer located at −15 kilobases, called the distal regulatory element (DRE). This enhancer contains binding sites for sex-determining region Y-box transcription factor 10 (SOX10) and microphthalmia-associated transcription factor (MITF); MITF is a transcription factor in pigment cells, and SOX10 is necessary for full enhancer activity in melanoma cells. Both factors activate the tyrosinase enhancer, although SOX10 does so directly, highlighting the contribution of SOX10 to the regulation of the *tyrosinase* gene in melanocytes [9]. Furthermore, chromosome conformation capture assays demonstrated that the DRE folds within the chromatin to physically contact the promoter region of the *Tyr* gene, facilitating transcriptional activation through the cooperation of SOX10 and MITF in both regulatory regions [8].

In humans, the expression of the *Tyr* gene is primarily controlled by the MITF, which binds to specific E-box motifs, particularly the M-box, an 11-base-pair sequence containing the consensus CATGTG motif, within the *Tyr* promoter to activate its transcription in melanocytes [10,11]. MITF also coordinates the expression of other melanogenic enzymes such as tyrosinase-related protein 1 (TYRP1) and dopachrome tautomerase (TRP-2), which participate in melanin biosynthesis and stabilize tyrosinase activity within melanosomes [12,13]. The human TYR protein is a type I transmembrane glycoprotein localized in the melanosomal membrane, where its luminal catalytic domain contains two copper ions that drive the hydroxylation and oxidation of L-tyrosine and L-DOPA [14]. Proper folding, glycosylation, and trafficking through the endoplasmic reticulum–Golgi–melanosome pathway are essential for its activity; mutations affecting these processes result in oculocutaneous albinism type I (OCA1) [15].

In *Agaricus bisporus* (button mushroom), it has been documented that the species has six tyrosinase isoenzymes (AbPPO1 to AbPPO6; Ab = *Agaricus bisporus*, PPO = polyphenol oxidase), each with different substrate affinities and catalytic rates, suggesting functional specialization in the fungus rather than a single generic enzyme. These isoenzymes are expressed from fungal mRNA and can be heterologously expressed for functional study [16]. At the structural level, mushroom tyrosinase can exist as a heterotetrametric complex composed of two heavy and two light subunits (*H*_2_*L*_2_ configuration) [17] and has latent regions that require activation (for example, by sodium sulfate to become functional) [18]. In terms of function, these isoenzymes participate in enzymatic darkening, fungal tissue defense, and phenolic compound management, rather than specialized pigmentation as in animals [19].

In plants, the enzymes known as polyphenol oxidases (PPOs) which include isoforms with tyrosinase activity are located predominantly in plastids or cellular vacuoles, and their genes typically form small multigene families whose expression is induced by stress signals, pathogens, or tissue damage [20]. These plant PPOs retain the binuclear copper center typical of animal tyrosinase but exhibit greater diversity in subcellular localization, substrate specificity, latency, and function; for example, they contribute to the formation of defensive pigments, cell-wall hardening, and browning after tissue wounding or cutting [21]. Functionally, in many species, PPO activity triggers the oxidation of phenols to quinones that subsequently polymerize, which contributes to the browning of fruits or plant tissues when they are damaged or exposed to oxygen [22].

Tyrosinase not only plays an important role in the biosynthesis of melanin, but it is also highly relevant in different fields. One of them is the food industry, since the catalytic product *o*-quinone is transformed into melanin through a series of reactions that cause the browning of fruits, vegetables, and seafood, primarily via an enzymatic browning reaction in which monophenols are hydroxylated to o-diphenols and then oxidized to o-quinones that polymerize into brown pigments [23].

This phenomenon leads to changes in color and flavor, decreasing the quality and nutritional value of the products, which in turn reduces food safety and consumer acceptance, consequently promoting food waste [24,25].

In the cosmetic industry, tyrosinase is the main therapeutic target for the development of depigmenting agents, since it catalyzes the rate-limiting steps of melanogenesis [26]. Its inhibition helps reduce melanin production and control hyperpigmentation disorders such as melasma, considered one of the most common pigmentary conditions [27], as well as senile lentigines, solar spots, or post-inflammatory hyperpigmentation [28], all of which significantly affect aesthetic appearance and quality of life [29]. These clinical signs of pigmentation can be improved by suppressing the activation of melanocytes—which are the cells in charge of skin pigmentation [6]—or by inhibiting the synthesis or transfer of melanin. Preventive strategies commonly include topical products and, more recently, dietary supplements. However, there is a growing use of skin-lightening products, such as hydroquinone, corticosteroids, and mercury-based compounds, which are prohibited due to their toxicity [27].

**Figure 5:**
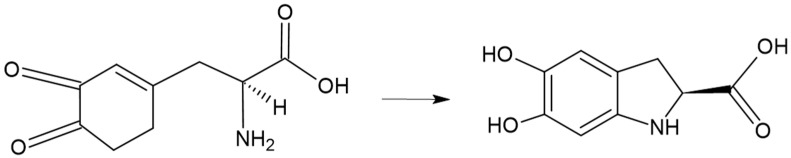
Non-enzymatic cyclization of dopaquinone to leucodopachrome and dopachrome. These spontaneous reactions lead to the formation of indolic intermediates that serve as precursors for eumelanin synthesis [34].

**Figure 6:**
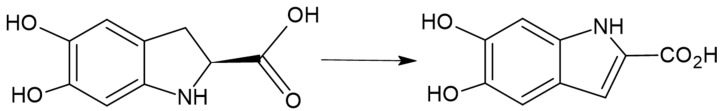
Dopachrome tautomerization catalyzed by dopachrome tautomerase (TRP-2/DCT). Dopachrome is converted into 5,6-dihydroxyindole-2-carboxylic acid (DHICA), a key intermediate in eumelanin biosynthesis [34].

**Figure 7:**
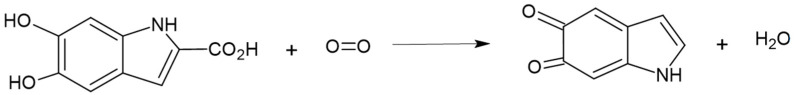
Oxidation of DHICA to indole-5,6-quinone-2-carboxylic acid (IQCA) catalyzed by tyrosinase-related protein 1 (TYRP1). TYRP1 catalyzes the oxidation of DHICA to IQCA, an indolic quinone intermediate containing a carboxylic acid group that contributes to eumelanin polymer formation [34].

**Figure 8:**
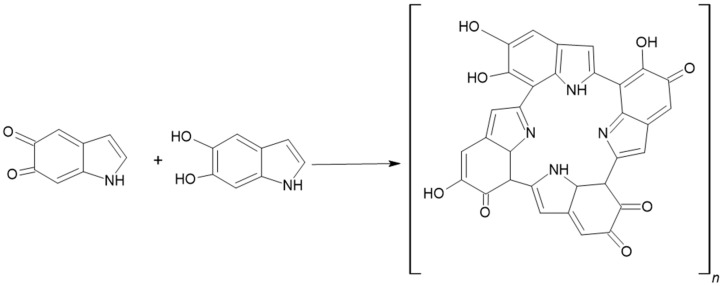
Auto-oxidation and coupling of indolic intermediates leading to eumelanin polymer formation. Indole-5,6-quinone (IQ) and 5,6-dihydroxyindole (DHI) undergo non-enzymatic oxidation and coupling reactions, resulting in heterogeneous eumelanin polymers [34].

Melanoma is a particularly aggressive form of skin cancer, arises from the transformation of these melanocytes, referring to the process by which normal pigment-producing cells acquire genetic mutations and biochemical alterations that disrupt the regulation of melanogenesis, where the complete chemical reaction is shown in Figure 3, Figure 4, Figure 5, Figure 6, Figure 7 and Figure 8, leading to uncontrolled proliferation, loss of cellular homeostasis, and ultimately tumor development [30]; this underscores the need to thoroughly understand the mechanisms associated with the regulation of melanogenesis [6]. Under physiological conditions, melanogenesis is a tightly regulated process that ensures the controlled accumulation of melanin, essential for protecting the skin against ultraviolet radiation [31]. However, when this regulation is disrupted, the excessive or abnormal synthesis of melanin can contribute to cellular alterations in melanocytes, favoring their transformation and the subsequent development of melanoma [30]. In this process, tyrosinase catalyzes the hydroxylation of L-tyrosine to L-DOPA and its subsequent oxidation to o-quinones, key intermediates in pigment formation. These reactions lead to the generation of dopachrome and initiate a cascade of biochemical transformations that culminate in melanin synthesis [32].

In addition, the inhibition of tyrosinase provides a therapeutic approach and can be considered more than just a cosmetic intervention; it offers a way to act directly on the biological root of pigmentary disorders. By blocking the enzyme responsible for initiating melanin synthesis, it becomes possible to normalize the overproduction of pigment that characterizes conditions such as melasma or post-inflammatory hyperpigmentation [35]. This approach is relevant because it does not simply mask the dark spots on the skin but intervenes in the pathway that drives their appearance [30]. Studies have shown that natural and microbial-derived compounds can reduce melanin formation by inhibiting tyrosinase activity, which positions this strategy as a promising path toward mechanism-based therapeutic modulation, rather than merely palliative treatments [29]. More generally, it has been proposed that this enzyme participates in oxidative processes associated with neurodegenerative diseases. In particular, studies have linked tyrosinase activity to the accumulation of neuromelanin and oxidative stress in dopaminergic neurons, processes strongly implicated in the pathogenesis of Parkinson’s disease [36]. Furthermore, emerging evidence suggests that tyrosinase-mediated oxidation of catecholamines could also contribute to neuronal damage observed in Alzheimer’s disease and other neurodegenerative conditions [37], highlighting its importance not only in dermatologic but also in biomedical research. This systematic review aims to analyze the inhibitor potential of microbially derived metabolites as a possible sustainable source of tyrosinase inhibitors with a particular emphasis on their biochemical diversity, inhibition mechanisms, and experimental evaluation strategies that may allow a comparison with conventional compounds currently employed in the health, cosmetic, and food industries. Previous reviews predominantly focus on plant-derived or synthetic compounds as tyrosinase inhibitors; this work specifically addresses microbial metabolites as an emerging and underexplored source of bioactive compounds. Furthermore, this review highlights the pronounced heterogeneity in experimental designs, enzyme sources, substrates, and activity expression units, which currently limits direct comparison among studies. By systematically identifying these methodological gaps and limitations, this review seeks not only to summarize existing evidence but also to provide a framework and recommendations for improving experimental standardization and translational relevance in future research.

## 2. Methods

### 2.1. Search Strategy

In accordance with PRISMA guidelines, a search of studies was conducted on the Scopus and Web of Science databases in March 2025. The equation used for Web of Science was ((“tyrosinase inhibitor” OR “inhibition of tyrosinase”) AND (“production” OR “biosynthesis” OR “accumulation” OR “secretion”) AND (“microorganism*” OR bacteria OR fungi OR yeast OR microalgae)) NOT (plant OR herbal OR botanical) NOT (human OR “Homo sapiens” OR mammal* OR animal* OR insect*), and for Scopus, it was TITLE-ABS-KEY (“tyrosinase inhibitor” OR “inhibition of tyrosinase”) AND TITLE-ABS-KEY (“production” OR “biosynthesis” OR “accumulation” OR “secretion”) AND TITLE-ABS-KEY (“microorganism*” OR bacteria OR fungi OR yeast OR microalgae) AND NOT TITLE-ABS-KEY (plant OR herbal OR botanical) AND NOT TITLE-ABS-KEY (human OR “Homo sapiens” OR mammal* OR animal* OR insect*).

### 2.2. Bibliography Selection

Based on the query equations provided earlier, the literature was leaked by using the inclusion and exclusion criteria. Inclusion criteria were (1) only article written in English, and (2) patents of metabolites, extracts, or culture-derived compounds from microorganisms, and (3) original research articles. Defined exclusion criteria were (1) review articles, systematic reviews, book chapters, and non-conventional academic sources, (2) metabolites obtained apart from microorganisms.

**Figure 9:**
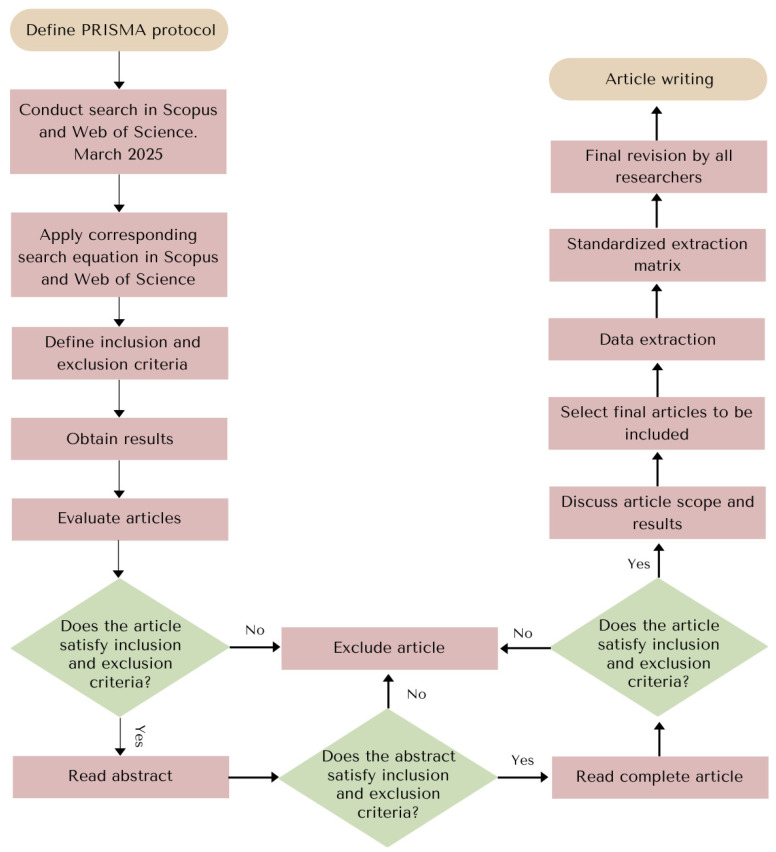
Flow diagram of the search strategy, screening process, and final article selection according to PRISMA guidelines. Pink boxes indicate the start and end points of the workflow, lilac boxes represent procedural actions, and green diamond-shaped boxes correspond to decision-making steps based on inclusion and exclusion criteria.

The scientific research articles were selected through an initial blind screening process developed by five researchers, based on the information provided in the titles and abstracts. A study was selected if at least three researchers agreed. The five researchers then read and discussed all of the articles in full and, after reading, only 11 were considered, together with two patents mainly focused on methodological approaches for obtaining microbial-derived compounds rather than on the characterization of microbial tyrosinase inhibitors. Data extraction was developed and reviewed by all five investigators through a standardized extraction matrix. Figure 9 shows the flow diagram of the developed methodology.

## 3. Results and Discussion

### 3.1. Selection Process and Selected Articles Overview

**Figure 10:**
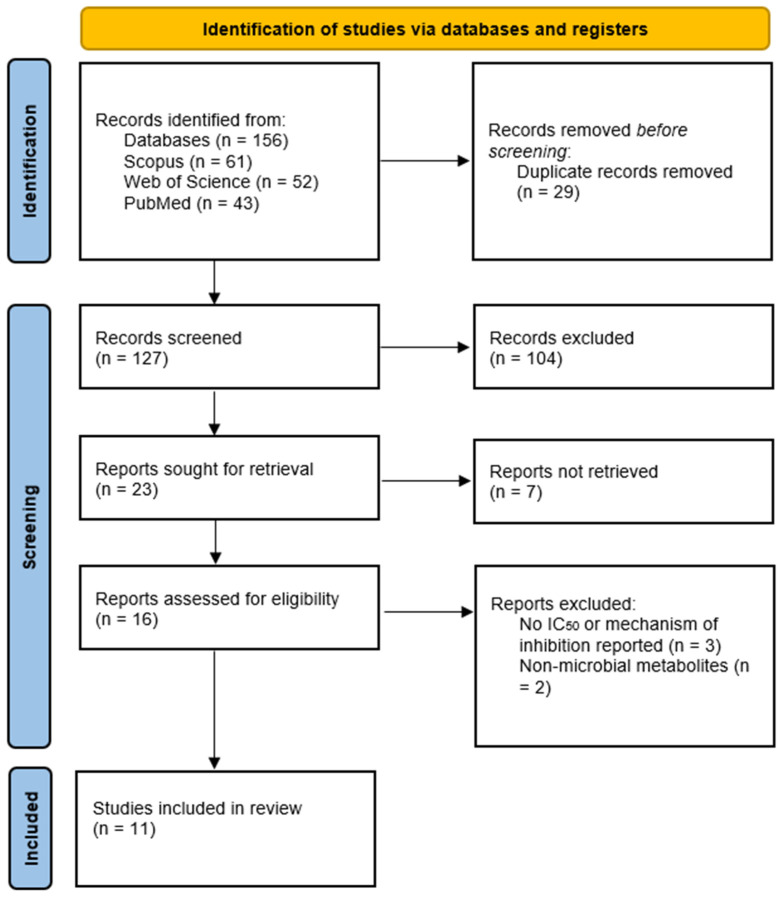
Identification, screening, and inclusion of studies for qualitative synthesis following PRISMA guidelines [38].

The initial search identified 156 articles. After removing 29 duplicates, 127 records were screened based on titles and abstracts. Of these, 104 were excluded for not meeting the inclusion criteria. Finally, 11 studies met all inclusion criteria and were incorporated into the qualitative synthesis. Figure 10 shows the flow diagram of the identification, screening, and included studies according to PRISMA guidelines. To contextualize the qualitative synthesis, Table 1 summarizes the main methodological characteristics of the selected studies, highlighting differences in enzyme sources, substrates, assay designs, and activity metrics reported that limit direct comparison of inhibitory potency.

**Table 1:** Comparative overview of methodological and biochemical heterogeneity among studies evaluating microbial tyrosinase inhibitors.

Enzyme Source	Substrate	Assay Type	Activity Metric	Key Methodological Remark	Reference
Mushroom tyrosinase	L-DOPA	In vitro enzymatic (colorimetric)	IC_50_ (mM)	Standard mushroom tyrosinase assay; no kinetic parameters or inhibition mechanism reported	[29]
Mushroom tyrosinase	α-glucosidase	In vitro enzymatic (dual-enzyme colorimetric)	IC_50_ (mM)	Mixed enzymatic system complicates attribution of inhibitory specificity toward tyrosinase	[39]
Potato tyrosinase	L-tyrosine	In vitro enzymatic (kinetic)	IC_50_ μM)	Non-competitive inhibition determined kinetically, but no structural or docking validation	[40]
Mushroom tyrosinase	L-tyrosine	In vitro enzymatic (colorimetric)	IC_50_ μM)	Activity reported for a mixture of structurally related compounds; individual contributions unresolved	[41]
Mushroom tyrosinase	L-tyrosine	In vitro enzymatic + molecular docking	Kinetic parameters (mM)	Competitive inhibition supported by docking, but enzyme source remains non-human	[30]
Mushroom tyrosinase	L-tyrosine	In vitro enzymatic + cellular + zebrafish	IC_50_ μM); gene expression	Combines enzymatic inhibition with transcriptional downregulation, improving biological relevance	[42]
Extracellular tyrosinase (*melC2*) in *Streptomyces avermitilis*	L-DOPA	Gene knockout + enzymatic oxidation assays	Qualitative activity change	Focuses on transcriptional regulation rather than direct enzyme inhibition	[43]
Mushroom tyrosinase	L-tyrosine	In vitro enzymatic (culture supernatant)	% inhibition	Crude extract; activity decreases over time, suggesting instability or degradation of active metabolites	[44]
*Streptomyces bikiniensis*-based melanin assay	Melanin biosynthesis pathway	Indirect bioassay	% inhibition	Indirect inhibition model; no isolated enzyme or defined substrate	[45]
Tyrosinase-related biofilm assay	Not specified	Biofilm inhibition assay	LOEC μg/mL)	Tyrosinase inhibition inferred indirectly; not a classical enzymatic assay	[46]
Mushroom tyrosinase	L-tyrosine	In vitro enzymatic (crude extract)	Qualitative inhibition	Absence of quantified activity metrics and lack of compound characterization	[47]

Table 1 highlights substantial methodological heterogeneity across the included studies, which currently limits robust quantitative comparison of microbial tyrosinase inhibitors. Although most entries evaluate inhibition using mushroom tyrosinase and in vitro colorimetric readouts with L-tyrosine or L-DOPA, several studies diverge in critical experimental features, including enzyme origin, assay format (enzyme kinetics vs. organismal melanin-biosynthesis or biofilm assays), and even the biological endpoint (direct enzymatic inhibition vs. transcriptional downregulation). These differences are reflected in the table itself, where outcomes are reported as IC_50_ (mM), IC_50_ (µM), IC_50_ (µg/mL), LOEC, percentage inhibition thresholds, or are not reported at all, making “potency ranking” across studies intrinsically uncertain without normalization.

A central source of variability is the enzyme model. The predominant reliance on commercially available *Agaricus bisporus* tyrosinase is experimentally convenient, yet it introduces translational caveats because fungal tyrosinase differs from the human enzyme in key biochemical features, for example, subcellular localization and oligomeric state, and the lack of routinely available purified human tyrosinase has historically constrained direct human benchmarking [48]. Consistent with this limitation, comparative work has shown that AbTYR and human TYR can respond differently to the same inhibitor, partly because the two proteins share low sequence similarity and exhibit distinct active-pocket features [49]. Therefore, activity values derived from mushroom tyrosinase should be interpreted as screening-level evidence rather than direct predictors of human efficacy, unless supported by additional human-relevant validation.

Heterogeneity is further amplified by substrate choice and functional readout. L-tyrosine-based assays probe monophenolase (cresolase) activity, whereas L-DOPA–based assays probe diphenolase (catecholase) activity, and inhibitors may not exert identical effects across these catalytic steps. This becomes especially relevant when studies combine enzymatic inhibition with broader biological outcomes such as reduced melanogenic gene expression, because the apparent “inhibition” may represent mixed contributions from enzyme-level and pathway-level regulation rather than a single binding mechanism.

Finally, the table underscores that inhibitory metrics are frequently reported under non-equivalent experimental conditions. Because IC_50_ values are condition dependent (notably on enzyme, substrate, and other assay parameters), direct comparisons across studies are only meaningful when conditions are closely matched or when parameters enabling mechanistic normalization, such as K_i_ under defined kinetics, are provided [50]. In addition, several entries cite comparator standards (commonly kojic acid) without reporting the comparator concentration or assay-matched reference values, limiting benchmarking utility. Collectively, Table 1 supports a cautious interpretation of “stronger” vs. “weaker” inhibitors across the literature and reinforces the need for improved reporting consistency, particularly standardized enzyme/substrate conditions, unified activity units (with molecular-weight normalization when applicable), explicit comparator benchmarks, and human-relevant confirmatory models, before firm translational or industrial claims can be made.

### 3.2. Microbial Metabolite Identification on Food, Cosmeceutical, and Health Industries

**Figure 11:**
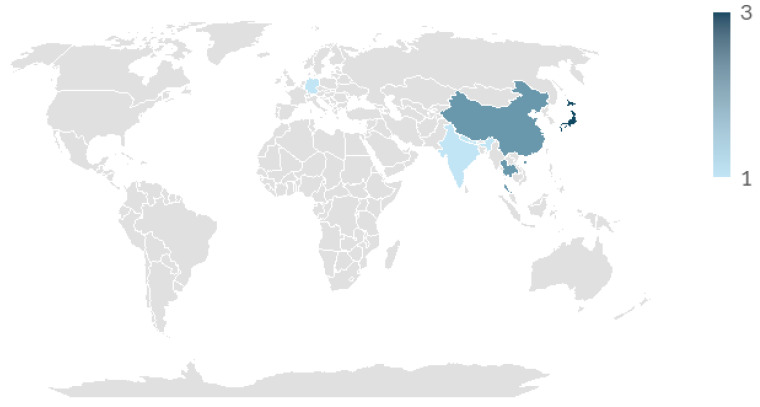
World distribution of investigations in degradation of microbial metabolites as tyrosinase inhibitors, where x-axis represents countries, and the y-axis represents the number of studies. Blue color intensification represents higher number of studies. Grey color means no studies were identified.

The systematic analysis of the literature review revealed a growing interest in investigating natural tyrosinase inhibitors. Publications on this topic began in 1995 and have significantly increased since the 2010′s. Geographically, the majority of articles are from Asian countries (Japan, China, India, Nepal, and Thailand), representing approximately 82% of the total, which reflects their strong research capacity and the high consumer demand for skin-lightening products [51]. Europe (Germany and Italy) accounts for the remaining 18%, highlighting regional efforts to explore microbial sources and biotechnological strategies as alternatives for tyrosinase inhibition. Figure 11 shows the world distribution of investigations explained before. This general increase is related to the expansion of the cosmeceutical and pharmaceutical industries and the search for safer alternatives to synthetic inhibitors [52] such as hydroquinone which are related to permanent leukoderma [53] and exogenous ochronosis [54]. Finally, 50% of the articles reported on cosmeceutical applications, 30% focused on the food industry, and the remaining articles focused on medical approaches.

Microbial sources include fungi, bacteria, and microalgae. Most of the studies are focused on filamentous fungi and actinobacteria; however, this reveals important gaps in the diversity of microorganisms explored. Yeasts and Gram-negative bacteria remain largely underrepresented despite their broad capacity for synthetizing bioactive compounds [55]. Despite archaea’s capacity for metabolite production under extreme conditions with high stability [56], investigations have not considered them. Principal isolations of microorganisms have been made in Asia, showing a clear limited exploration of microbial diversity in megadiverse countries such as Brazil and Colombia where microbial richness remains largely untapped. All these gaps highlight promising opportunities for research in novel tyrosinase inhibitors beyond the traditional focus.

The inhibitors have been identified as organic compounds ranging from indole derivatives, polyketides, peptides, and complex secondary metabolites. Extracts of microbial culture supernatants were also reported, although many studies remain limited to crude or partially purified preparations. Specifically, three studies (30%) used crude extracts, two (20%) used partially purified fractions, and the remaining five (50%) used purified or chemically characterized compounds. One of the best characterized compounds is indole-3-carbaldehyde, obtained from the fungal strain YL185 and purified from extracellular fluid. It was tested in enzyme assays—achieving an IC_50_ of 1.3 mM—and in B16 melanoma cells, showing a reduction in cellular melanin in a mammalian melanocyte-derived cell line directly relevant for depigmentation claims [29]. This inhibitory potency, although moderate, remains relevant compared to kojic acid (IC_50_ ≈ 0.4 mM), the current reference standard.

To synthesize the information obtained from the reviewed studies, Table 2 summarizes the principal characteristics of microbial-derived tyrosinase inhibitors, including the producing organisms, type of compound, inhibition assays, and reported activities. This comparative overview highlights the biochemical diversity of microbial metabolites involved in tyrosinase inhibition, ranging from indole derivatives and phenolic acids to peptides and triterpenoids. It also reveals the predominance of in vitro enzymatic assays employing mushroom tyrosinase and colorimetric methods with L-DOPA or L-tyrosine as substrates. Additionally, the table outlines the limited number of studies that report kinetic constants (IC_50_ or Ki values) and the scarcity of data on inhibition mechanisms or structure–activity relationships. Together, these results provide a basis for identifying trends, methodological gaps, and potential directions for future research on microbial tyrosinase inhibitors.

**Table 2:** Microbially derived compounds with reported tyrosinase inhibitory activity under experimental conditions and their potential applications.

Inhibitor (Compound)	Microbial Source	IC_50_ Value	Mechanism of Inhibition	Comparative Reference	Application	Reference
Indole-3-carbaldehyde	Fungus YL185	1.3 mM	Not specified	Kojic acid (unreported inhibitory concentration)	Cosmetics, food, medical	[29]
*p*-coumaric acid	*Spirulina* spp.	52.71 ± 3.01 mM	Reversible mixed-type inhibition (Cu^2+^ interactions)	Kojic acid and ascorbic acid (unreported inhibitory concentration)	Food, cosmetics	[39]
Methyl lucidenate F	*Ganoderma lucidum*	0.03223 mM (Ki = 0.01922 mM) ^a^	Non-competitive inhibition	Kojic acid (18.4 µM)	Cosmetics, skin whitening	[40]
Kyonggic acids (1–4)	*Massilia kyonggiensis*	0.166–0.355 mM ^a^	Not specified	Kojic acid (220 µM)	Cosmetics	[41]
YL-6 peptide	*Schizophyllum commune*	0–8 mM (kinetic)	Competitive inhibition (H-bond and hydrophobic site binding)	Kojic acid (16.3 µM)	Cosmetics, therapeutic	[30]
AK-12 peptide (AILQSYSAGKTK)	*Synechococcus* sp.	0.4897 mM (monophenolase), 0.7656 mM (diphenolase) ^a^	Competitive inhibition; downregulation of MITF, TYR, TYRP1, TRP-2	Kojic acid (30.6 µM)	Medical, cosmetic	[42]
Daidzein/3ODI	*Streptomyces avermitilis*	Gene regulation (non-enzymatic inhibition)	Gene-regulated (non-enzymatic inhibition)	Extracellular tyrosinase (*melC2*) knockout	Medical	[43]
Culture supernatant (uncharacterized metabolite)	*Trichoderma* sp. H1-7	Not specified	Unknown (activity decreased after 3 days)	Kojic acid (Unreported inhibitory concentration)	Food, cosmetic	[44]
Crude microalgal extracts	Marine microalgae (28 strains)	<30% inhibition	Indirect inhibition (melanin pathway)	Not reported	Food, cosmetic	[45]
Ciclo-L-Trp-L-Ala and related dipeptides	*Eurotium chevalieri* MUT 2316	0.001 µg/mL (3.632 × 10^−6^ mM) ^b^ (LOEC)	Not specified	No control used	Marine antifouling	[46]
Crude culture extract	*Kitasatospora* sp. SBSK430	Not reported	Not specified	Kojic acid (unreported inhibitory concentration)	Medical	[47]

^a^ In order to have greater homogeneity of units that allow comparison of the results in all articles, the unit has been changed in those results that are in other units reported in the articles. ^b^ Molecular weight of Ciclo-L-Trp-L-Ala is 275.31 Da, which was taken as average to convert the LOEC given in the original article to being available of comparison.

Table 2 highlights the diversity of microbial tyrosinase inhibitors in terms of both origin and inhibitory efficiency. Reported IC_50_ values range from the micromolar to millimolar scale, showing substantial variability across compounds and microbial sources. The most potent inhibitors include *Ciclo-L-Trp-L-Ala* obtained from the marine fungus *Eurotium chevalieri* (LOEC 0.001 µg/mL) and Methyl lucidenate F from *Ganoderma lucidum* (IC_50_ = 32.23 µM), both corresponding to well-characterized metabolites with clearly defined chemical identities. Notably, these compounds differ markedly in their structural classes, as Ciclo-L-Trp-L-Ala is a low-molecular-weight cyclic dipeptide, whereas methyl lucidenate F is a triterpenoid, suggesting that molecular size, functional groups, and overall polarity may influence their interaction with tyrosinase and contribute to differences in inhibitory potency. In contrast, crude culture supernatants such as those from *Trichoderma* sp. or marine microalgae extracts demonstrated moderate inhibition, which supports the need for purification and structure elucidation to accurately identify the active compounds responsible for the observed activity.

The comparison of analytical conditions also reveals important methodological inconsistencies. While most studies used mushroom tyrosinase and L-DOPA as substrate, only a few included reference inhibitors such as kojic acid for activity benchmarking. Additionally, the expression units for IC_50_ or inhibition percentage differ among studies, hindering direct potency comparisons and reflecting the lack of standardized protocols for tyrosinase inhibition assays.

A second relevant pattern relates to biological validation. Although the majority of assays rely exclusively on enzymatic inhibition models, some recent studies have expanded their analyses to include in vitro melanocyte systems or in vivo zebrafish pigmentation models, indicating progress toward biologically relevant testing. In this regard, bioactive peptides such as YL-6 from *Schizophyllum commune* and AK-12 from *Synechococcus* demonstrate not only enzyme inhibition but also downregulation of melanogenesis-related genes, confirming dual biochemical and transcriptional mechanisms.

Finally, from an application perspective, the inhibitory activity reported does not always correlate with suitability for cosmetic, food, or medical purposes. Some compounds, such as *p*-coumaric acid from *Spirulina*, exhibit moderate inhibition (IC_50_ ≈ 52.7 mM) but combine antioxidant and α-glucosidase inhibition properties, suggesting multifunctional potential for food preservation and nutraceutical use. Overall, the data indicate a gradual transition from crude extract evaluations toward the isolation of structurally defined metabolites and mechanistic validation through molecular docking or kinetic analysis. Nonetheless, persistent variability in assay design and the limited use of human tyrosinase models remain key barriers for accurately determining inhibitory potency and advancing translational applications.

Mechanistically, most inhibitors act via direct competitive or non-competitive inhibition of tyrosinase. From the analyzed studies, two compounds were reported as competitive inhibitors (peptides YL-6 and AK-12), one as non-competitive (Methyl lucidenate F), and five did not specify their inhibition mechanism, while one involved transcriptional regulation instead of enzyme inhibition [56]. For the YL-6 peptide, molecular docking analyses suggested binding at the active center of tyrosinase, including interactions with catalytic residues and the binuclear copper ions, supporting a competitive mode of inhibition through restriction of substrate access to the active site. In contrast, although the AK-12 peptide was classified as a competitive inhibitor based on kinetic data, the original study does not provide detailed information regarding its exact binding site or specific interactions with copper ions. Similarly, for methyl lucidenate F, the non-competitive inhibition was established through kinetic analyses, but no structural or molecular evidence was reported to define its binding region or to explain potential conformational effects on the enzyme. Typically, enzymatic assays are carried out through colorimetric techniques using L-tyrosine and L-3,4-dihydroxyphenylalanine (L-DOPA) and mushroom tyrosinase [29,39,40]. Strategies beyond enzyme inhibition have also been explored. The authors of [43] evaluated the gene expression of extracellular tyrosinase (*melC2*) in *Streptomyces avermitilis*. The researchers deleted the *melC2* gene to demonstrate that the enzyme participates in degrading daidzein-derived compounds such as 7,3′,4′-trihydroxyisoflavone (3′ODI), which are susceptible to conversion into quinones and melanin-like pigments. This study showed that microbial tyrosinases not only influence melanogenesis but also direct enzymatic oxidation via transcriptional regulation of P450 enzymes and related factors, confirming the intervention with pigmentation pathways.

However, reliance on non-human tyrosinase remains a critical limitation for achieving translational relevance; thus, results obtained for fungal tyrosinase cannot necessarily be extrapolated to human tyrosinase due to structure differences and kinetics. An additional limitation is the scarce and non-standardized reporting of inhibitor structural/physicochemical descriptors (e.g., molecular weight, polarity, functional groups) and cytotoxicity endpoints, which limits structure–activity relationship (SAR) interpretation and prevents robust safety profiling of candidate inhibitors. Before advancing to in vitro or in vivo studies in human systems, it is essential to incorporate computational and experimental approaches that strengthen the human relevance of the inhibitor, as outlined below.

Molecular docking and molecular dynamics: Docking allows for the prediction of the binding orientation and estimated affinity of candidate ligands to human-derived or homology-modeled tyrosinase, while molecular dynamics (MDs) simulations test the stability of ligand–protein complexes over time, revealing conformational fluctuations and energetic behaviors [57].

Homology modeling: When the crystal structure of human tyrosinase is not available, one can use a related enzyme with known structure (e.g., mushroom tyrosinase, or human TYRP1) as a template to build a 3D model of human tyrosinase (hTYR) and then refine it for para docking/MD purposes. Currently, some studies use homology modeling followed by virtual screening to discover new tyrosinase inhibitors [58].

Site-directed mutagenesis (directed mutagenesis): This is a widely used molecular technique for introducing specific point mutations into selected residues of the target gene, with the aim of generating protein variants under controlled modifications [59]. This strategy is useful to test hypotheses derived from structural models or docking by comparing catalytic activity, kinetic constants, and inhibitor affinity between the wild-type enzyme and its mutant versions; one can determine which residues are essential for substrate or inhibitor binding, or for the catalytic function of the enzyme itself.

Combining computational modeling with site-directed mutagenesis is a well-established approach in enzymology to validate predicted functional sites and to quantify the energetic contribution of individual amino acids in catalysis or transition-state stabilization [60,61]. Although direct applications to human tyrosinase are still limited, this combined strategy has been successfully applied in other metalloenzymes to identify residues responsible for metal coordination and substrate recognition insights that could be translated to refine homology-modeled or docking-based hypotheses for human tyrosinase. Therefore, directed mutagenesis could support this study by experimentally validating critical residues involved in copper binding or catalysis, guiding the rational design of more robust and selective inhibitors with higher translational potential under physiological conditions.

Virtual screening of natural product libraries: This involves applying molecular docking (often combined with filters for Absorption, Distribution, Metabolism, Excretion, and Toxicity (ADMET), Quantitative Structure–Activity Relationship (QSAR), and physicochemical properties) to large libraries of natural metabolites to prioritize promising candidates. For instance, some studies employed machine learning combined with docking on natural product libraries and approved inhibitors to reduce the search space in the identification of new tyrosinase inhibitors [62]. More specifically, a recent study used the Natural Product Activity and Species Source Database (NPASS) in conjunction with docking and active-learning approaches to discover novel tyrosinase inhibitors [63].

In food-related contexts, tyrosinase inhibition is primarily linked to the control of enzymatic browning in fruits and vegetables, a process that compromises both sensory attributes and nutritional value [29]. For example, indole-3-carbaldehyde isolated from the fungus YL185 is a potential candidate to mitigate browning reactions in food matrices, given the central role of tyrosinase in color deterioration during storage and processing [29]. In addition, phenolic compounds extracted from *Spirulina*, particularly *p*-coumaric acid, have demonstrated tyrosinase inhibitory activity (IC_50_ ≈ 52.7 mM), along with α-glucosidase inhibition, pointing to a dual role as browning control agents and multifunctional additives for functional foods [39].

On the other hand, in cosmetic applications, microbial tyrosinase inhibitors have been widely investigated as safer and more sustainable alternatives to conventional depigmenting agents. The reviewed studies reveal that bioactive compounds derived from fungi, algae, and bacteria can effectively regulate melanogenesis through enzymatic and genetic pathways. At the same time, Indole-3-carbaldehyde isolated from the extracellular fluid of the fungus YL185 significantly inhibited mushroom tyrosinase (IC_50_ = 1.3 mM) and reduced melanin synthesis in B16 melanoma cells, demonstrating preliminary depigmenting potential in experimental models [29]. Likewise, marine-derived peptides have gained increasing attention for their mildness and biocompatibility. The peptide AK-12 from the marine microalgae *Synechococcus* exhibited dual inhibition of mono- and diphenolase activities (IC_50_ = 489.7 and 765.6 μM, respectively), alongside downregulation of MITF, TYR, TYRP1, and TRP-2 genes in melanoma cells and zebrafish models, without cytotoxic effects [42]. Similarly, the YL-6 peptide derived from the split-gill mushroom (*Schizophyllum commune*) competitively inhibited tyrosinase (IC_50_ = 3.97 mM for monophenolase and 6.75 mM for diphenolase activity) and suppressed melanogenic protein expression in B16F10 cells, highlighting its formulation-oriented potential, supported by cellular and zebrafish models, but still requiring validation in human skin systems [30]. Moreover, *p*-coumaric acid isolated from *Spirulina* demonstrated mixed-type reversible inhibition of tyrosinase and strong copper-binding capacity, supporting its dual cosmetic and nutraceutical relevance [39].

Additionally, in the medical and pharmaceutical context, microbial tyrosinase inhibitors are being investigated not only for their depigmenting potential but also for their ability to modulate oxidative stress and melanogenic signaling pathways associated with pathological hyperpigmentation and melanoma. *Streptomyces avermitilis* has been proposed as a microbial model to understand tyrosinase-mediated oxidation processes in metabolic and signaling contexts. The deletion of its extracellular tyrosinase gene (*melC2*) altered the expression of the enzyme CYP105D7 and electron-transfer proteins (Fpr and Fdx), reducing the hydroxylation of isoflavones but preventing metabolite degradation—an effect that reveals the broader role of tyrosinase in redox balance and cellular bioactivation [43]. Moreover, the recently identified kyonggic acids produced by *Massilia* spp. exhibited tyrosinase inhibition comparable to that of arbutin (IC_50_ = 166–176 μM) and structural similarities to dehydroamino acids with antioxidant potential, suggesting their relevance as lead scaffolds for further pharmacological development, rather than ready-to-use therapeutic agents [41].

Kojic acid is widely recognized as an excellent tyrosinase inhibitor [63], which is why its obtention has been investigated for years. One of the eldest patents for improved methods of extracting kojic acid from microbial sources was developed in 1965. Although high yields were achieved, the use of heavy metals compromises the sustainability of the method, as well as the ability to maintain controlled chemical conditions [64]. In contrast, a Japanese patent describes a process of obtaining kojic acid from microorganisms through sublimation, which increases coloring stability and yield, specifically for cosmetic applications as an external use drug for suppressing melanization with no need for heavy metals or strong acids and remarkable dermatologic safety [65]. However, recent evaluations highlight safety concerns for kojic acid, which has been associated with adverse cutaneous reactions—including contact dermatitis, sensitization, erythema, and redness—prompting interest in alternative tyrosinase-inhibiting scaffolds with better tolerability [66].

Overall, the reviewed evidence demonstrates that microbial metabolites possess considerable potential as sustainable sources of tyrosinase inhibitors, with promising applications in cosmetic, food, and biomedical industries. Recent studies, such as those on *Massilia kyonggiensis* (kyonggic acids) and *Spirulina platensis* (*p*-coumaric acid), revealed inhibitory activities comparable to or exceeding those of arbutin or kojic acid, yet with indications of lower toxicity and favorable structural features, as inferred from in vitro and model-organism studies [39,41]. Likewise, peptide compounds such as YASILL, derived from *Schizophyllum commune*, exhibited competitive inhibition toward human tyrosinase and a marked reduction in melanogenesis in vitro without cytotoxicity, reinforcing their cosmetic and therapeutic applicability [30]. However, most studies remain limited to assays, with scarce clinical trials and little methodological standardization, which restricts extrapolation of the findings to commercial or pharmacological formulations. Consequently, although there is clear biotechnological potential, further work on stability, bioavailability, and in vivo validation is required to consolidate microbial inhibitors as realistic alternatives to the synthetic compounds currently used in cosmetic and dermatological markets.

## 4. Conclusions

This systematic review provides a focused and critical synthesis of microbially derived tyrosinase inhibitors, an area that has received comparatively limited attention in previous reviews largely centered on plant-derived or synthetic compounds. Beyond compiling reported inhibitory activities, this work highlights the pronounced heterogeneity in experimental designs, enzyme sources, substrates, and activity reporting metrics, which currently limits direct cross-study comparison and translational interpretation. This manuscript demonstrates that microorganisms represent a promising and sustainable source of natural tyrosinase inhibitors with potential relevance to cosmetic, food, and biomedical research. Fungal, bacterial, and microalgal species have yielded structurally diverse metabolites—including indole derivatives, phenolic acids, peptides, and triterpenoids—capable of modulating melanogenesis through both enzymatic and transcriptional mechanisms. Compared to conventional inhibitors such as kojic acid and arbutin, microbial compounds exhibit comparable or superior inhibition profiles while offering improved biocompatibility and environmental safety, although these observations remain largely dependent on in vitro experimental conditions. However, most studies remain limited to in vitro assays employing mushroom tyrosinase, with scarce data on human enzymes or in vivo validation, thus constraining translational relevance. This limitation underscores the need for improved experimental standardization, including harmonized assay conditions and activity reporting metrics. Standardization of assay conditions, structural elucidation of active metabolites, and mechanistic modeling using human tyrosinase are essential next steps to overcome these limitations. Future research should also explore omics-guided discovery approaches, molecular docking, and mutagenesis-based validation to strengthen the understanding of enzyme–inhibitor interactions. Overall, microbial metabolites stand out as a largely untapped biotechnological resource for the rational eco-friendly development of tyrosinase inhibitors with potential future clinical relevance, provided that current methodological and translational gaps are systematically addressed.

## Data Availability

Not applicable.
